# Supplementary figures and images for: 2HybridTools, a handy software to facilitate clone identification and mutation mapping from yeast two-hybrid screening
Source: PeerJ. 2019 Jul 3;7:e7245. doi: 10.7717/peerj.7245 (PMC6612259; doi:10.7717/peerj.7245)

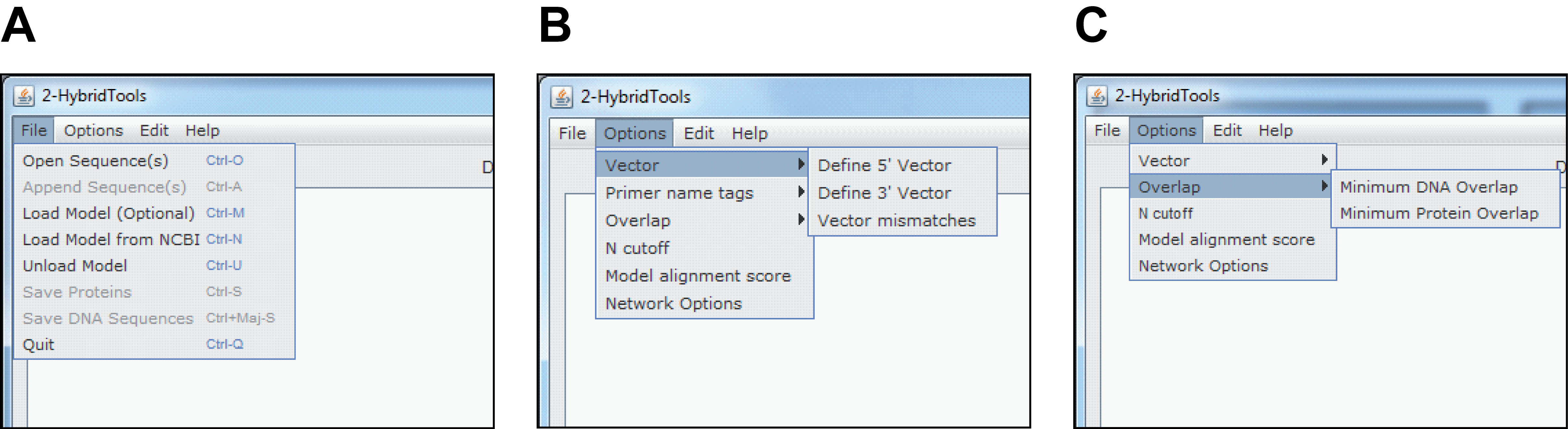

Supplement: Figure S1 — Drop down menus showing (A) file options, (B) vector options: 5’ and 3’ vector sequences, mismatches and (C) overlap options for assembly of forward and reverse sequencing products. [file peerj-07-7245-s001.png]

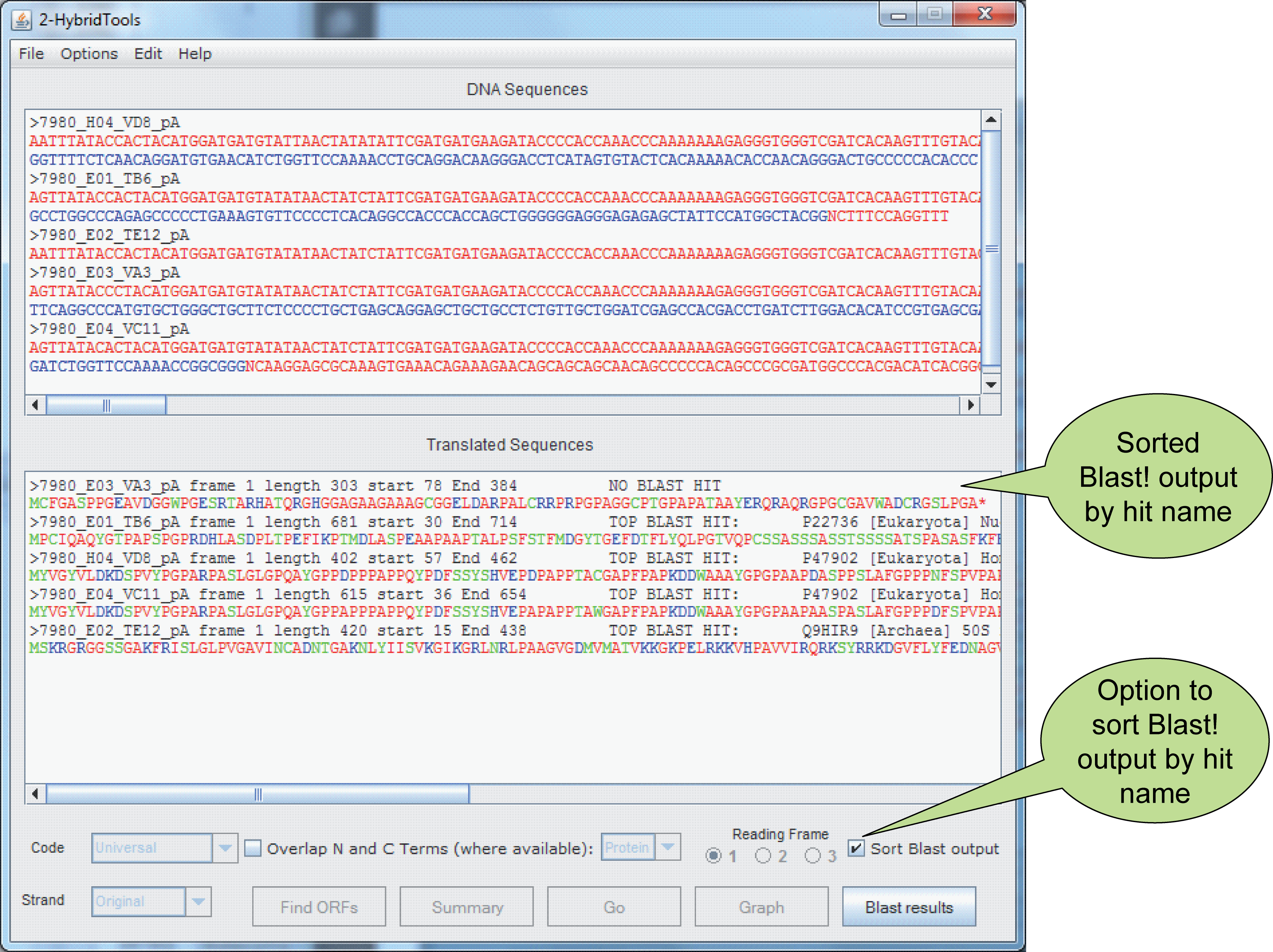

Supplement: Figure S2 — Alphabetical sorting is enabled by the Sort Blast output checkbox. [file peerj-07-7245-s002.png]

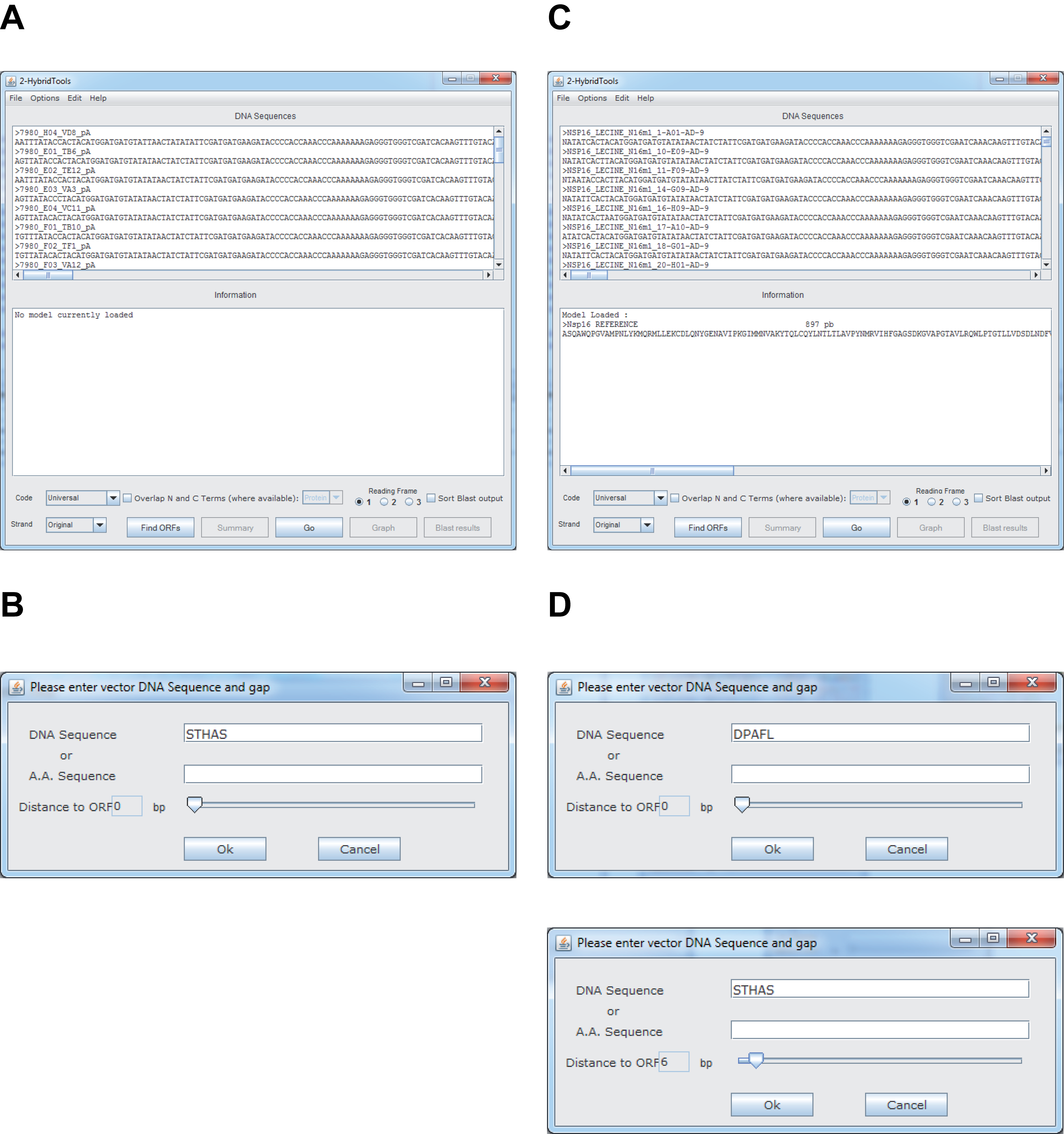

Supplement: Figure S3 — (A, B) Y2H mode, loading sequences (A), and setting 5’ vector tag sequence as STHAS, with ORF starting directly after tag sequence (B). (C,D) RY2H mode, loading sequences as well as reference model (C), and setting 5’ vector tag sequence as STHAS, with ORF starting 6 residues after tag sequence (D, top); setting 3’ vector tag as DPAFL, with ORF starting ending directly before tag sequence (D, bottom). [file peerj-07-7245-s003.png]
